# PRSice: Polygenic Risk Score software

**DOI:** 10.1093/bioinformatics/btu848

**Published:** 2014-12-29

**Authors:** Jack Euesden, Cathryn M. Lewis, Paul F. O’Reilly

**Affiliations:** MRC Social, Genetic and Developmental Psychiatry Centre, Institute of Psychiatry, Psychology and Neuroscience, King’s College London, London, United Kingdom

## Abstract

**Summary:** A polygenic risk score (PRS) is a sum of trait-associated alleles across many genetic loci, typically weighted by effect sizes estimated from a genome-wide association study. The application of PRS has grown in recent years as their utility for detecting shared genetic aetiology among traits has become appreciated; PRS can also be used to establish the presence of a genetic signal in underpowered studies, to infer the genetic architecture of a trait, for screening in clinical trials, and can act as a biomarker for a phenotype. Here we present the first dedicated PRS software, PRSice (‘precise'), for calculating, applying, evaluating and plotting the results of PRS. PRSice can calculate PRS at a large number of thresholds (“high resolution”) to provide the best-fit PRS, as well as provide results calculated at broad *P*-value thresholds, can thin Single Nucleotide Polymorphisms (SNPs) according to linkage disequilibrium and *P*-value or use all SNPs, handles genotyped and imputed data, can calculate and incorporate ancestry-informative variables, and can apply PRS across multiple traits in a single run. We exemplify the use of PRSice via application to data on schizophrenia, major depressive disorder and smoking, illustrate the importance of identifying the best-fit PRS and estimate a *P*-value significance threshold for high-resolution PRS studies.

**Availability and implementation:** PRSice is written in R, including wrappers for bash data management scripts and PLINK-1.9 to minimize computational time. PRSice runs as a command-line program with a variety of user-options, and is freely available for download from http://PRSice.info

**Contact:** jack.euesden@kcl.ac.uk or paul.oreilly@kcl.ac.uk

**Supplementary information:** Supplementary data are available at *Bioinformatics* online.

## 1 Introduction

The polygenic model of human phenotypes has long been hypothesized, but only in recent years have the results from genome-wide association study (GWAS) revealed that much of the genetic basis for most complex traits comprises small effects of hundreds or even thousands of variants. For clinical outcomes, this polygenic effect can be considered a genetic liability to disease risk. While prediction of phenotype from an individual’s genetic profile is compromised by this polygenicity, the application of polygenic risk scores (PRS) has shown that prediction is sufficiently accurate for a number of applications.

A PRS for an individual is a summation of their genotypes at variants genome-wide, weighted by effect sizes on a trait of interest. Effect sizes are typically estimated from published GWAS results, and only variants exceeding a *P*-value threshold, *P*_T_, are included (Dudbridge, 2013). Since even large GWAS achieve only marginal evidence for association for many causal variants, PRS are usually calculated at a set of *P*-value thresholds, e.g. $P_{T}=1\times 10^{-5},1\times 10^{-4},\ldots ,0.05,0.1,\ldots ,0.5$. A common application of PRS is to test for shared genetic aetiology between traits. Here PRS on the *base phenotype* are calculated, using GWAS results, in individuals from an independent data set, and these are used as predictors of the *target phenotype* in a regression (see Supplementary Note S1). This technique was first applied by the International Schizophrenia Consortium (2009), demonstrating that genetic risk for schizophrenia (SCZ) is a predictor of bipolar disorder. This study also acted as a proof-of-principle for PRS, showing that PRS based on thousands of common variants genome-wide, including many with no effect and using effect size estimates from published GWAS, can provide a reliable indicator of genetic liability. This has motivated several other applications, including polygenic Mendelian Randomisation (Hung *et al.*, 2014), where causality of potential intermediate phenotypes in a disease pathway can be tested (Ehret *et al.*, 2011), use of PRS as biomarkers, and the enrolment of clinical trial participants according to risk (Hu *et al.*, 2013).

Here we describe the first dedicated and fully automated software package for the application of PRS - PRSice. PRSice has a high-resolution option that returns the best-fit PRS, has a flexible set of user options intended to capture current standard practices in PRS studies and the different applications of PRS, and produces plots for inspection of results. We also perform a simulation study to estimate a *P*-value significance threshold for high-resolution PRS studies.

## 2 Overview of PRSice

PRSice has been developed to fully automate PRS analyses, substantially expanding the capability of PLINK-1.9 (Chang *et al.*, 2014). A key feature of PRSice is that it can calculate PRS at any number of *P*-value thresholds (*P*_T_) and can thus identify the most predictive (precise) threshold. It requires only GWAS results on a *base phenotype* and genotype data on a *target phenotype* as input (base and target phenotype may be the same); it outputs PRS for each individual and figures depicting the PRS model fit at a range of *P*_T_. PRSice allows users to include or remove SNPs in linkage disequilibrium, handles genotyped and imputed data, and can calculate ancestry-informative dimensions for use as covariates. These features integrate R code with computations performed in PLINK-1.9 and extensive bash scripts to minimize computational time. PRSice is a command-line program that allows users to apply a large number of PRS, under different parameter settings or across multiple base and target traits. In addition to the standard approach, there is an option to use summary statistics for the target as well as the base data, using the gtx package (Johnson, 2013). For future updates of PRSice, see the website: http://PRSice.info.

## 3 Results

**Fig. 1.**
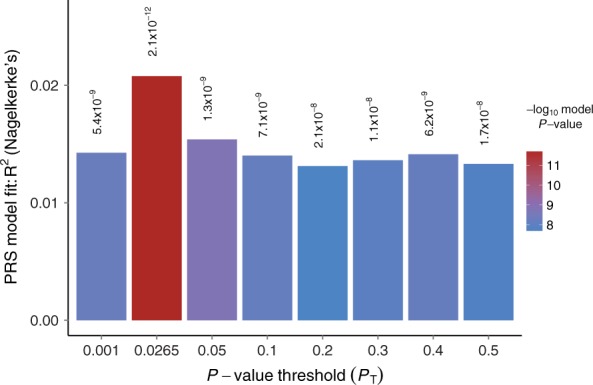
Bar plot from PRSice showing results at broad *P*-value thresholds for Schizophrenia PRS predicting MDD status. A bar for the best-fit PRS from the high-resolution run is also included

**Fig. 2.**
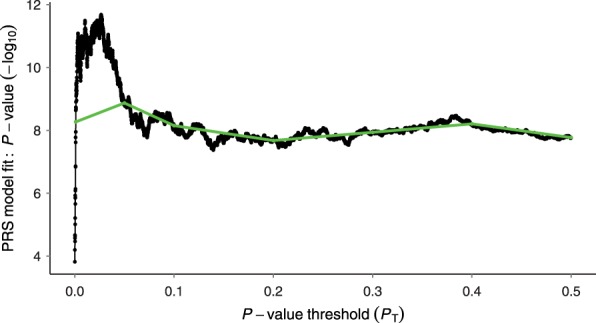
High-resolution PRSice plot for SCZ predicting MDD status. The thick line connects points at the broad *P*-value thresholds of Fig.1

Here we illustrate the use of PRSice to test for shared genetic aetiology between traits. We first investigate the genetic relationship between schizophrenia (SCZ) and major depressive disorder (MDD), both known to be complex and comorbid. We apply PRSice to replicate the finding by Smoller *et al.* (2013) that SCZ PRS can predict MDD status, using the RADIANT-UK MDD case-control data set (Supplementary Note S2, Lewis *et al.*, 2010). Applying PRSice, we remove SNPs in linkage disequilibrium and include principal components to control for population structure. We find significant evidence that SCZ PRS predict MDD status, and under the approach of only testing PRS at several broad *P*-value thresholds find the most predictive threshold at *P*_T_ = 0.05 (Fig. 1). Next we repeat the analysis using high-resolution PRS (Supplementary Note S3) and find the most predictive PRS at $P_{T}=0.0265$ (Fig. 2). The PRS at $P_{T}=0.05$ explains 1.5% of the variation in MDD (Nagelkerke *R*^2^; $P=1.3\times 10^{-9}$) whereas the high-resolution best-fit PRS explains 2.1% ($P=2.1\times 10^{-12}$) and is based on 5252 fewer SNPs (12148 rather than 17400).

Next we apply PRSice to two tobacco-related phenotypes from the TAG consortium (Thorgeirsson *et al.*, 2013) and the RADIANT-UK MDD data. These analyses reveal, for the first time, shared genetic aetiology between the dichotomous trait ‘ever smoked’ and MDD, but not between smoking consumption, as a quantitative trait, and MDD (Supplementary Fig. S1). In the former, high-resolution scoring again produces a substantially different best-fit PRS than that from broad *P*_T_, in terms of model fit, significance and number of SNPs included (Supplementary Fig. S1b).

Under high-resolution PRS in particular, multiple tests are performed and so the *P*-value of the best-fit PRS will be inflated. Therefore, we perform a permutation study utilizing the SCZ and MDD data described above, and estimate an adjusted significance threshold for the best-fit PRS of *P* = 0.004 (Supplementary Note S4). Prior to a more extensive study, we suggest a more conservative significance threshold of *P* = 0.001 if using the best-fit PRS for association testing in PRS studies.

## 4 Discussion

Here we have described our PRSice software, illustrating its use with three PRS studies. We have illustrated the potential benefit of obtaining the best-fit PRS and have estimated a corresponding significance threshold. There is great potential for the future application of PRS in genetics: for gaining insights into the genetic architecture of a trait by comparing observed PRS with theoretical expectations across a range of *P*_T_ (International Schizophrenia Consortium, 2009), for assessing the genetic overlap of a trait(s) across populations, for use as biomarkers, as instrumental variables, or even to provide evolutionary insights (Berg and Coop, 2014). The PRS approach, and PRSice software, could be extended to test the effects of copy number variants, epigenetic markers and more. We believe that PRSice can simplify PRS studies greatly, expand the application of PRS and aid the implementation of best-practice in PRS studies.

## Funding

MRC studentship (to JE), EU FP7 no. 279227(PsychDPC), and the NIHR Biomedical Research Centre at SLaM and KCL.

*Conflict of Interest*: none declared.

## Supplementary Material

Supplementary Data
