# Supplementary material for: PRSice: Polygenic Risk Score software
Source: Bioinformatics. 2014 Dec 29;31(9):1466–8. doi: 10.1093/bioinformatics/btu848 (PMC4410663; doi:10.1093/bioinformatics/btu848)
Supplement: Supplementary Data [file supp_31_9_1466__index.html]

PRSice: Polygenic Risk Score software — PRSice: Polygenic Risk Score software — PRSice: Polygenic Risk Score software — Supplementary Data 

# PRSice: Polygenic Risk Score software

## Supplementary Data

files

**Files in this Data Supplement:**

- Supplementary Data - zip file
