# Supplementary material for: PRSice: Polygenic Risk Score software
Source: Bioinformatics. 2014 Dec 29;31(9):1466–8. doi: 10.1093/bioinformatics/btu848 (PMC4410663; doi:10.1093/bioinformatics/btu848)
Supplement: Supplementary Data [file supp_btu848_suppl_data.zip › PRSice_Supplementary_REVISION.pdf]

Supplementary Material

PRSice: Polygenic Risk Score software

Jack Euesden, Cathryn M. Lewis, Paul F. O'Reilly
MRC Social, Genetic and Developmental Psychiatry Centre, King's College London

Supplementary Note 1: Polygenic Risk Scores applied between traits

Polygenic risk scores are calculated across *n* individuals from the 'target phenotype' data set using a list of *m* SNPs, the genotypes of which have some effect (or not) on the 'base phenotype'. The base and target phenotype may be the same, if assessing the shared genetic overlap of a phenotype between samples/populations. These genotype effects can be estimated from a univariate regression of base phenotype on each SNP, such as from a genome-wide association study (GWAS). In such a GWAS, for a SNP *i*, where $i = 1, 2, ..., m$, a *P*-value, P_i, is calculated for the association between the SNP genotypes, $G_{i,j} = \{0,1,2\}$ for individual *j* where $j = 1, 2, \dots, n$, and the phenotype. Under the usual additive assumption made in GWAS, a corresponding effect size is estimated, by β_i, for the effect of a unit increase in genotype, G_{ij}, on the phenotype.

SNPs are generally selected for inclusion in a polygenic risk score based on the degree of evidence, according to *P*-value, for their association with the base phenotype in a GWAS – SNP *i* will be included in a PRS if P_i is smaller than a threshold, P_T. PRS are typically calculated at a number of different *P*-value thresholds, P_T.

At threshold P_T, the PRS for individual *j* can be calculated as:

$$PRS_{P_T,j} = \sum_{i=1}^{m} \beta_i G_{i,j}$$

PRS, which we see here are based on effect size estimates relating to the 'base phenotype', are calculated across all individuals giving *n* scores per threshold, P_T. The association between these PRS and the target phenotype can then be evaluated in an appropriate regression model (depending on the data type of the target phenotype, eg. linear regression if the phenotype is continuous).

This can be repeated across *q* *P*-value thresholds, P_T, and the model fit of the regression of target phenotype on PRS compared.

In real data there is usually some missing genotype data, unless genotypes have already been imputed. PLINK-2 imputes any missing data according to mean allele frequencies.

Supplementary Note 2: Data sets analysed

Base phenotype data set

In the main analysis we used the publicly available results from the largest Schizophrenia GWAS to date (Psychiatric Genomics Consortium (2014)) for the base phenotype data set. In the additional analyses on Smoking behaviour and MDD, we used GWAS results from the Tobacco and Genetics (TAG) consortium on two phenotypes as the base phenotype: the binary phenotype 'ever smoked' and the quantitative trait smoking consumption, as measured by average number of cigarettes smoked per day (Thorgeirsson et al. (2013)). In each case we removed any SNPs with poor imputation quality (info score < 0.7).

Target phenotype data set

We used genotype data from the RADIANT-UK consortium (Lewis et al. (2010)), a sample of 1624 depression cases and 1588 psychiatrically screened healthy controls, for the target phenotype data set for each PRS analysis. These were genotyped on the Illumina HumanHap 610 QuadBead Chip. Quality control was performed, removing individuals with missingness > 1%, abnormal heterozygosity, conflicting sex and reported gender and those of non-European ancestry or close relatedness based on principal components. SNPs with MAF < 1% and SNPs not in HWE ($P < 1x10^{-5}$) were also removed.

We used the first two eigenvectors calculated using EIGENSTRAT as ancestry informative dimensions, to adjust for population structure. Linkage disequilibrium (LD) was accounted for by selecting the SNP in the base phenotype data set with the lowest discovery *P*-value in a sliding window of 250kb, only retaining variants with a pairwise LD $r^2 < 0.1$, according to LD calculated in the target data set. We performed high-resolution scoring by testing every threshold between $P_T = 0.0001$ and $P_T = 0.5$ at increments of 0.00005. This produces 9999 thresholds.

Supplementary Note 3: High-resolution polygenic risk scoring

High-resolution polygenic risk scoring, as performed in PRSice, calculates PRS at a large number of evenly spaced *P*-value thresholds, between a minimum and maximum bound. For the analysis here, we use a lower bound of $P = 0.0001$ and an upper bound of $P = 0.5$, and increments of 0.00005. This generates 9999 thresholds. Assuming that there are ~100k SNPs in approximate linkage equilibrium with $P < 0.5$, 10 SNPs would be added per threshold if *P*-values were uniformly distributed across SNPs. In practice, GWAS results will be enriched for small *P*-values, due to association with the base phenotype and due to *P*-value informed clumping preferentially extracting SNPs with small *P*-values. Therefore, the number of SNPs included at each threshold will decrease at larger *P*-value thresholds. This high-resolution approach enables us to identify the best-fit PRS to a high degree of approximation; the true best-fit PRS can only be identified by testing PRS at every possible P_T, but we instead test them at high-resolution in order to reduce total computational time substantially with negligible loss in accuracy.

Supplementary Note 4: Multiple testing correction

High-resolution polygenic scoring fits a large number of regression models, as described above, and so the 'multiple testing problem' should be addressed when evaluating the significance of the best-fit PRS. Currently, uncorrected alpha thresholds of 0.05 are routinely used to assess the significance of PRS. Under high-resolution a small number of SNPs are added to the model at each new *P*-value threshold. Thus, the resulting PRS is likely to be very similar to the previous, especially once a large number of SNPs are already included, so there is high correlation between the multiple tests performed. Therefore, a simple Bonferroni correction or similar for the number of tests performed will produce an overly conservative adjustment for the multiple testing.

We performed three permutation studies to estimate an appropriate significance threshold that controls the family-wise error rate at 0.05 and accounts for the multiple tests performed in a high-resolution PRSice analysis. We calculated PRS repeatedly at high-resolution, using the GWAS results on Schizophrenia from the Psychiatric Genomics Consortium as base data and RADIANT-UK genotype data on MDD as target data (see Supplementary Note 2), under the null hypothesis of no association with the target phenotype by permuting case-control status in the MDD data set. We used data from chromosome 19, which should reflect genetic data across the genome, and permuted MDD case-control status in the RADIANT-UK data set 10000 times. As above, we performed clumping on the SNPs to remove the effects of SNPs in LD and adjusted for population structure with two principal components. In this way we obtained an empirical distribution for the *P*-value of the best-fit PRS. In order to understand the effect of sample size on this distribution, we repeated our permutation study in 1000, 2000 and 3000 individuals randomly sampled from the target data. These results indicated that an alpha threshold of 0.004 must be applied to high-resolution best-fit PRS in order to ensure a false-positive rate below 0.05 (table S1). Prior to an extensive study to estimate a more reliable significance threshold for high-resolution PRS, we suggest a more conservative significance threshold of $P = 0.001$.

**Sample Size**	**Empirical Significance Threshold**
1000	0.0042
2000	0.0042
3000	0.0046

Table S1: Empirical significance thresholds calculated from permutation, estimating the required significance threshold to interpret the results of high-resolution scoring, across different target data set sizes.

Supplementary Figure S1

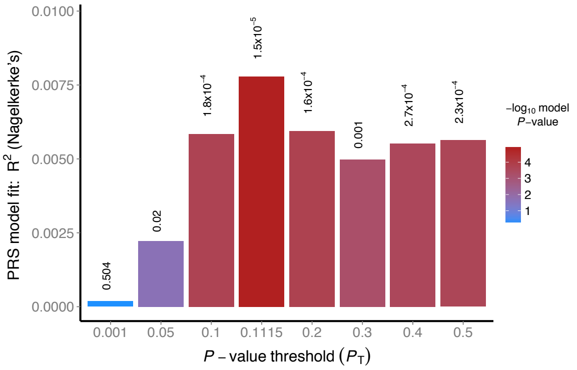

Figure S1a: 'Ever smoked': PRS using GWAS from the Tobacco and Genetics (TAG) consortium for 'ever smoked' as base phenotype data (N = 74053), and the RADIANT-UK MDD data as target phenotype data. SNPs in linkage equilibrium, adjusting for population structure using two principal components, show substantial evidence for shared genetic aetiology between smoking and MDD.

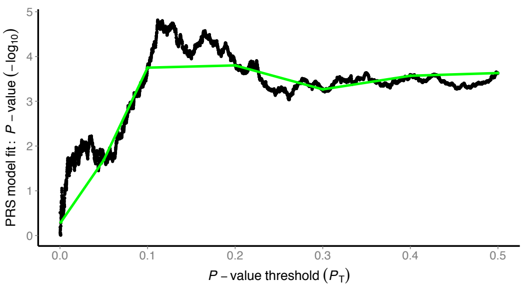

Figure S1b: 'Ever smoked': High-resolution PRS for 'ever smoked' predicting MDD status (see Fig. S1a). The high-resolution best-fit PRS is at P_T = 0.1115, while that based on broad thresholds on is P_T = 0.2.

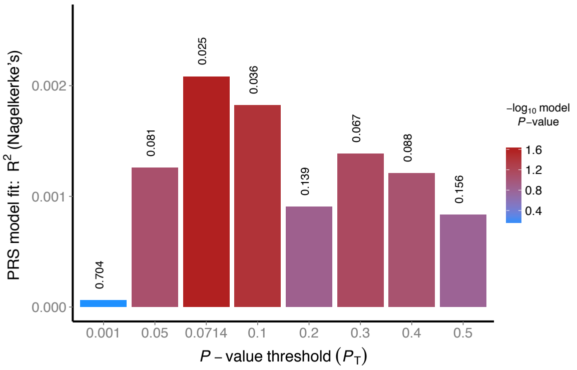

Figure S1c. Number of Cigarettes Smoked per day: Genetic risk of smoking more cigarettes as a quantitative trait, predicting MDD. This demonstrates no evidence for shared genetic aetiology between the two phenotypes, since the *P*-value of best-fit PRS (calculated from the high-resolution PRS) is > 0.001 (see Supp. Note 4).

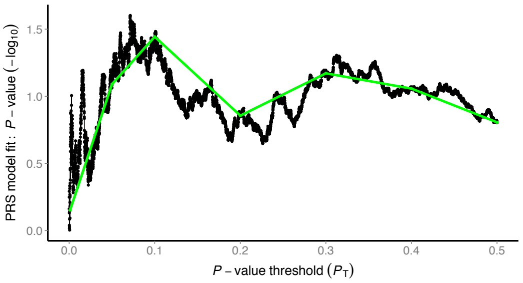

Figure S1d. Number of Cigarettes Smoked per day: High-resolution PRS for number of cigarettes smoked predicting MDD status. These high-resolution scores show that the results from the broad P-value thresholds of Figure S1c are not false negatives owing to the small number of thresholds considered.

Supplementary References

Lewis, C. M., Ng, M. Y., Butler, A. W., Cohen-Woods, S., Uher, R., Pirlo, K., . . . McGuffin, P. (2010). Genome-wide association study of major recurrent depression in the U.K. population. *American Journal of Psychiatry, 167*(8), 949-957. doi: 10.1176/appi.ajp.2010.09091380

Psychiatric Genomics Consortium. (2014). Biological insights from 108 schizophrenia-associated genetic loci. *Nature, 511*(7510), 421-427. doi: 10.1038/nature13595

Thorgeirsson, T. E., Gudbjartsson, D. F., Sulem, P., Besenbacher, S., Styrkarsdottir, U., Thorleifsson, G., . . . Stefansson, K. (2013). A common biological basis of obesity and nicotine addiction. *Translational Psychiatry, 3*. doi: 10.1038/tp.2013.81
